# Incorporating Stud Attachments in the Bar Design for an Implant-Supported Overdenture

**DOI:** 10.1155/2024/2818034

**Published:** 2024-06-19

**Authors:** Dimokritos Papalexopoulos, Christos Partalis, Panagiotis Lampropoulos, Ioli-Ioanna Artopoulou, Nikitas Sykaras

**Affiliations:** Department of Prosthodontics School of Dentistry National and Kapodistrian University of Athens, Zografou, Greece

## Abstract

Bone resorption following tooth loss might compromise retention, stability, and support of conventional removable prostheses, and for this reason, implant-supported overdentures are suggested as a viable alternative for completely edentulous patients. Bars, telescopic attachments, or stud attachments have been used to provide retention through a different mechanism of action based on specific design characteristics. The purpose of this report is to thoroughly describe the applied protocol for the fabrication of an implant overdenture supported by two bars incorporating stud attachments. A 67-year-old male patient presented to the Postgraduate Clinic of the National and Kapodistrian University in Athens seeking dental rehabilitation. The remaining teeth were characterized with poor prognosis, mainly due to their periodontal status. The proposed treatment plan included the placement of four implants in the maxilla and two implants in the mandible and the fabrication of implant-supported overdentures. The diagnostic stages revealed adequate prosthetic space that would enable the fabrication of a bar substructure for the maxillary overdenture. To combine the benefits of bars and stud attachments, two bars with four attachments were fabricated. Evaluation of the delivered prosthesis revealed adequate retention, support, and stability achieved with minimal palatal coverage. Patient's reported satisfaction and quality of life were increased. Recall appointments at one, six, and twelve months did not reveal any adverse effects or patient's complaints. According to the present case report, different types of attachments may be used after careful study of each case. More studies are needed to report on different aspects of the chosen treatment plan.

## 1. Introduction

For many years, conventional complete dentures were the only available treatment for edentulous patients [1, 2]. Tooth loss is accompanied by a series of deleterious effects. Edentulism is associated with nutritional deficits and functional and aesthetic concerns regarding masticatory ability, speech, and appearance [3]. There is also a serious impact on general health and quality of life [4]. However, residual ridge resorption impedes retention and stability of complete dentures, thus undermining adaptation to this type of prosthesis [5].

The McGill consensus established implant-supported overdentures as the first option for the restoration of the edentulous mandible [6], thereby changing a considered alternative to the gold standard for such cases [7]. Since then, they have been utilized for the restoration both of edentulous and partially edentulous patients with high survival rates [8]. The placement of 2-4 implants to support an overdenture provides a cost-effective treatment modality that improves retention, stability, chewing efficiency, and patient satisfaction [9].

Implant-supported overdentures are supported both by the residual alveolar ridge mucosa and the attachments placed on the osseointegrated implants [10]. Different attachment systems have been utilized for the retention of implant-supported overdentures [11]. Ball, stud, telescopic, bar, and clip attachments are the most common retention systems used in order to support an implant overdenture [12]. The selection of the best attachment system for each case should be made after thorough study and critical analysis. Retentive capacity, ridge arch shape, degree of residual ridge resorption, available prosthetic space, dimension and angulation of the placed implants, patient's needs, costs, treatment complexity, and postinsertion maintenance are some of the main factors that should be taken into consideration [13].

Stud attachments are widely used due to the simplicity of their use [14]. Low vertical height, minimum interarch space requirements, facilitation of oral hygiene, and ease of manipulation are also some of the characteristics of this type of attachment [15].

Bars provide the advantage of splinting which is crucial in case of implants with limited dimensions or unfavorable angulations [16]. This type of attachment has high survival rates and contributes to better force distribution and inhibition of lateral displacing forces, thus offering adequate stability [17]. On the other hand, there are several drawbacks including higher costs, technical sensitivity, and hygiene-related issues such as gingival hyperplasia [18].

Selection of the most appropriate attachment may be challenging for the dental clinician [19]. An ideal attachment should combine the advantages of both stud and bar attachments. However, such a system is not available. The purpose of this case report is to describe the steps followed for the rehabilitation of a patient with implant-supported overdentures with bars incorporating stud attachments.

## 2. Case Report

A 67-year-old male patient presented to the Postgraduate Prosthodontic Clinic of the National and Kapodistrian University of Athens. The patient had been receiving antiplatelet medication (clopidogrel) for the past 12 months due to an ischemic stroke. Even though he was accompanied by his son, the patient was fully independent.

**Figure 1:**
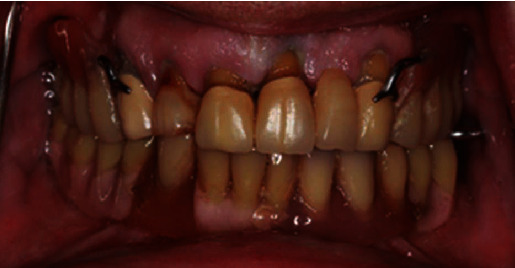
Initial situation.

Concerning his dental status, the patient reported lack of stability and retention of his existing removable partial denture (RPD) and asked for a prosthesis of improved retention and stability. Clinical examination revealed caries related to the fixed partial denture and the RPD lacked retention and stability while exerting lateral forces upon the abutment teeth. The edentulous mandible was rehabilitated with a conventional complete denture (Figure 1).

The maxillary anterior teeth were characterized with poor prognosis. At first, initial impressions were made, and the diagnostic casts were mounted according to jaw registrations. After communication with the physician to acquire information concerning the status of the underlying disease, the extraction of the remaining teeth took place, and the existing partial denture was modified to a complete denture and delivered as a transitional prosthesis in order to restore the masticatory ability [20].

**Figure 2:**
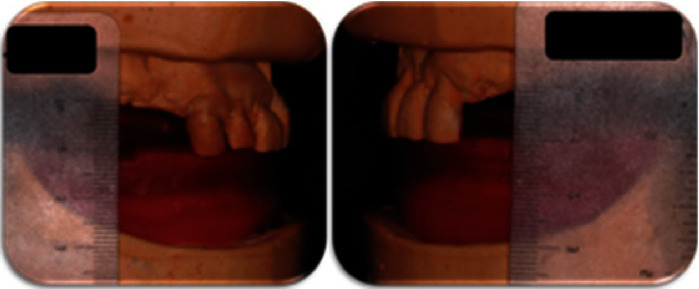
Restorative space available in the maxilla.

After 6 months, the patient had fully recovered and returned for the definitive restorations. The proposed treatment plan included the fabrication of implant-supported overdentures. Given the available prosthetic space measured at the mounted casts, a bar attachment was planned (Figure 2).

Initially, the patient was referred for a cone beam computed tomography (CBCT) in order to evaluate the bony substrate before implant placement [21]. Acrylic copy dentures with radiographic markers were used. After thorough analysis of the CBCT images, the placement of four implants in the maxilla and two in the mandible was decided.

**Figure 3:**
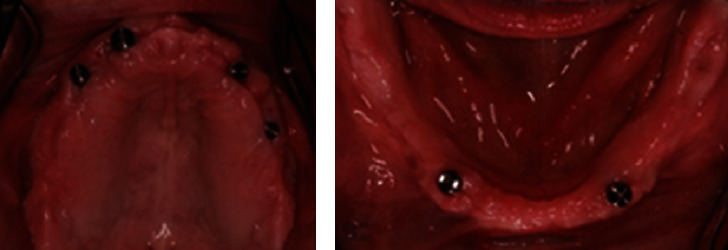
Implant dispersion regarding (a) the maxilla and (b) the mandible.

Four months after placement, the implants were uncovered, and healing abutments were placed (Figure 3). Two weeks later, preliminary impressions were made for custom trays to be fabricated. The acrylic custom trays (Triad, Consolidated Metal Products, Germany) were tried intraorally and adjusted. Even though retention is mainly provided by the attachments in the case of implant overdentures, border molding was performed with thermoplastic compound (Compound impression Stick, Kerr, KerrHawe S.A., Switzerland) to record mobile mucosa.

Open tray impression copings were placed on the maxillary implants and checked radiographically for their fit [22]. The impression copings were splinted and the splint was segregated and connected again with low-shrinkage acrylic resin (Pattern Resin, GC International AG, Luzern, Switzerland) to increase impression accuracy [23]. The custom tray was tried intraorally and confirmed that there was no interference with the copings or the splinting material during insertion. The impression of the maxilla was made with a combination of medium and low-viscosity polyvinylosiloxane (Zhermack, Zhermack GmbH, Italy). Due to the presence of some undercuts, the mandibular impression was made with a custom tray, and polysulfide material (Permplastic Regular, Kerr, KerrHawe S.A., Switzerland) was chosen because of its elasticity, after border molding.

**Figure 4:**
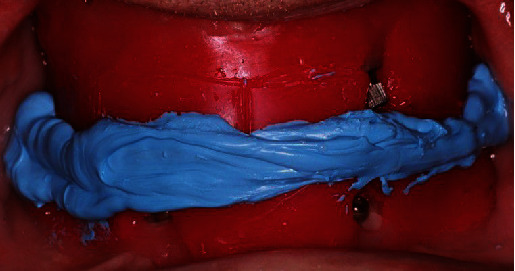
Centric relation record with screw-retained record base for the maxilla.

Record bases with occlusal rims were fabricated. The maxillary record base was screw retained in one of the four implants for better accuracy during the jaw registration appointment. Midline and canine positions were marked on the maxillary rim. Vertical dimension of occlusion was determined after measurement of the vertical dimension at rest, with a 4 mm difference between them. Centric relation was recorded at the previously defined vertical dimension with an occlusal registration polyvinylsiloxane (Variotime, Kulzer GmbH, Germany) (Figure 4).

**Figure 5:**
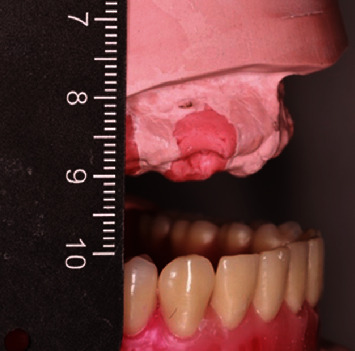
The presence of adequate restorative space for the construction of a bar substructure was verified.

A set-up trial appointment of the anterior teeth was performed. After approval of the midline, shape, form, shade, and arrangement, a set-up trial was performed for all teeth. Function, aesthetics, and phonetics were evaluated. Vertical dimension and centric relation were also verified. At this stage, the presence of adequate restorative space for the construction of a bar substructure was verified at the mounted casts (Figure 5).

**Figure 6:**
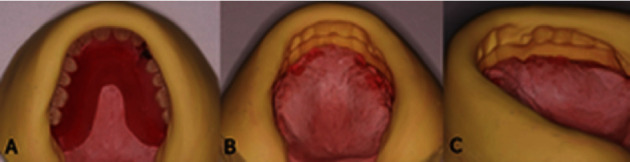
(a) Prosthetic space availability verification through matrices fabricated from the diagnostic set-up. Space should be evaluated both (b) horizontally and (c) vertically.

With the set-up trial placed on the working casts, silicone matrices (Oxasil, Kulzer GbH, Germany) were fabricated (Figure 6). The cast-matrix set-up enables available space measurements. In the case described, limited space was available at the central incisors area, so two separate bars were fabricated, in order to avoid a bulky prosthesis in the anterior region. More restorative space was available posterior to lateral incisors. This, along with the patient's desire for enhanced retention and easier maintenance, due to his medical record, led to the placement of two stud attachments on each bar in order to compensate for the uneven placement of implants, since in the case of bars, the retentive elements do not have to coincide with implant positions.

This configuration offers a self-aligning design with customizable levels of retention. Moreover, maintenance is easier than the traditional bar and clip design, including prosthesis activation after wear of the attachments. The selected attachments have a minimum vertical height, thereby not depriving the prosthesis from vital space for the other components.

**Figure 7:**
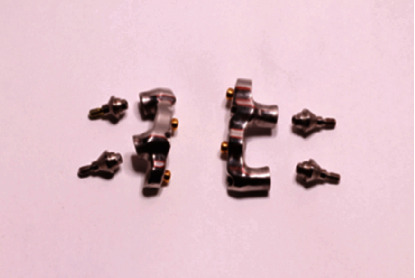
Space measurements should take into account implant abutments, bar height, and stud attachment height.

Space should be measured from the implant platform. In cases of implants with different inclinations or with increased gingival height, as the one described, adequate space for intermediate abutment placement must be present. The latter along with bar height and stud attachment height comprises the minimum vertical space needed for the overdenture substructure (Figure 7).

**Figure 8:**
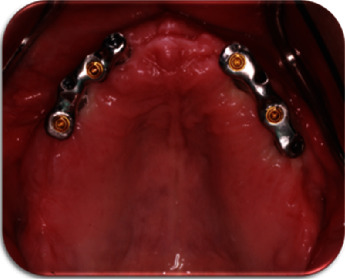
Bar design: stud attachments do not coincide with implant positions.

The two bars were fabricated so that enough space was present for the additional prosthetic components while complying with the minimum vertical height defined by the thread of the selected stud attachments that ensure their retention within the bar. Thread sockets have been made with the aid of a surveyor to ensure parallelism and a specific path of insertion. The bar-stud attachment configurations were evaluated both clinically and radiographically for the passivity of fit (Figure 8). Metal frameworks with the acrylic teeth set-up and their adaptation on the underlying bars were checked intraorally. Function, aesthetics, and phonetics were also evaluated.

At the delivery appointment, the implant abutments and the bars were positioned and torqued according to the manufacturer's instructions. The maxillary overdenture had been activated at the laboratory. The mandibular overdenture had sockets corresponding to the two implants in order to be intraorally activated. The presence of adequate space for the activation was verified after the insertion of the stud attachments and placement of the overdenture with low-viscosity polyvinylsiloxane (Zhermack, Zhermack GmbH, Italy) inside the sockets.

**Figure 9:**
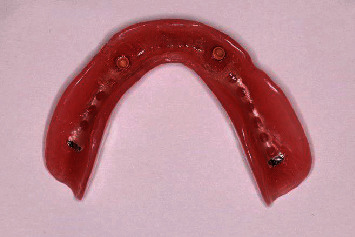
Intaglio view of the mandibular denture after removal of the excess material used at the activation process.

Since the patient should remain in centric occlusion during the activation process, low-viscosity acrylic resin (Pattern resin, GC International AG, Luzern, Switzerland) was chosen because of the limited setting time and was placed within the notches of the connected part. A thicker amount was placed inside the sockets on the intaglio surface of the overdenture. After the complete setting of the material, the overdenture was removed, excess material was trimmed, and the area was thoroughly polished (Figure 9).

**Figure 10:**
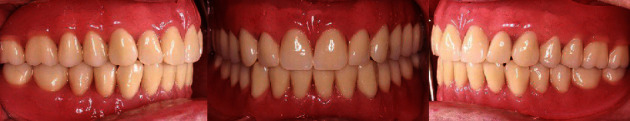
Intraoral view of the dentures at the delivery appointment.

Both the dentures were thoroughly inspected before the delivery (Figure 10). Instructions regarding the proper use and hygiene of the dentures were given to the patient. A recall appointment was scheduled after one week.

**Figure 11:**
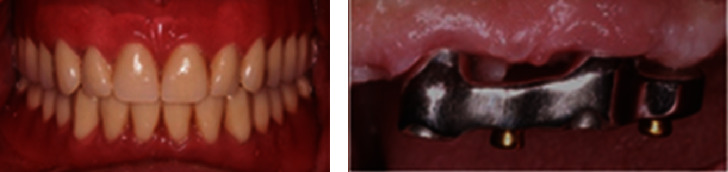
(a) Intraoral view at the 1-year recall. (b) Correct design and oral hygiene have led to the prevention of gingival hyperplasia.

At the recall appointment, the patient was satisfied with the overdentures, and no complaint regarding their use was expressed. Instructions regarding hygiene were repeated, and the patient was asked to return every 6 months for recall appointments. The 1-year recall appointment proved that careful exercise of oral hygiene on a daily basis has prevented the development of gingival hyperplasia (Figure 11).

## 3. Discussion

The described case is the first one to present a step-by-step protocol for the rehabilitation of an edentulous patient with overdentures supported by bars incorporating stud attachments, a treatment plan that has not drawn much attention in the literature. This treatment modality is aimed at combining the advantages offered by stud attachments regarding simplicity of use, easy maintenance procedures, and low costs and bars in terms of increased stability, implant splinting, and better force distribution [15, 17].

A critical point in the described treatment option is the management of the restorative space. Utilization of a bar has a vertical space requirement of at least 13 mm while stud attachments are less space demanding (9-12 mm) [24]. In this case, the stud attachments used offered the advantage of minimum vertical height. The available horizontal dimension should also be measured and taken into account during treatment planning and decision-making since there are some special bar designs, such as the Hader bar, that demand 10-12 mm of space [25].

Studies have underlined that the conversion of a conventional removable prosthesis into an implant-supported one can benefit patients with increased chewing efficacy, improved aesthetics, satisfaction, and quality of life [26]. Implant-supported overdentures comprise a restorative solution that combines improved function compared to conventional dentures and lower costs and surgical interventions as opposed to full-arch implant retained fixed prostheses [27].

Innovative technologies and materials have been incorporated into the implant-supported overdenture fabrication protocol, such as polyether-ether-ketone (PEEK). PEEK is a biocompatible, nonallergic, rigid, radiolucent, white polymer with low plaque affinity [28] and elastic modulus that is close to human bone [29]. Some recent studies have reported on the use of PEEK as a bar fabrication material with promising results [30] that must be further investigated. Another aesthetic material incorporated in many dental applications is zirconia [31], a biocompatible ceramic material with wear resistance and high mechanical properties. According to a crossover study, patient satisfaction was superior with zirconia than with cobalt-chromium bars in terms of psychological preference, appearance, time, hygiene, undergoing procedures, recommended procedures, and the overall experience [32]. However, the use of these materials demands novel technologies and dental laboratories with expertise and is more costly than the use of materials mentioned in this report.

Regarding the edentulous mandible, some researchers have proposed the concept of a single-implant-retained overdenture for both the treatment plan and surgical procedures to be simplified and costs to be reduced [21, 33]. A recent systematic review and meta-analysis showed that there are no differences between one- and two-implant-supported overdentures when it comes to prosthetic complications, oral health-related quality of life, and marginal bone loss within five years of function [34]. The supporters of this treatment modality have stated that it has the potential to become the new minimum standard.

To the best of our knowledge, this is the first report to describe a step-by-step protocol for the rehabilitation of an edentulous patient with overdentures supported by bars incorporating stud attachments. The described treatment plan offers a combination of the advantages that describe the use of bars and stud attachments. On the other hand, such a configuration is technically challenging and demands experience and skills from both the dental clinician and the dental technician. The presence of the described components and the required clinical and laboratory procedures result in the elongation of both the treatment time and costs.

## 4. Conclusion

This case describes the steps followed in the fabrication workflow of an implant overdenture supported by a bar incorporating stud attachments. This treatment plan is aimed at combining the benefits of the two attachment types concerning retention, stability, and ease of removal, facilitating oral hygiene and repair. In order to reap the benefits of this restorative choice, careful execution of the clinical steps and flawless communication between the clinician and the dental laboratory are needed. The 1-year recall has justified the applied treatment plan in terms of patient satisfaction and tissue response.

## Data Availability

Data supporting this research article are available from the corresponding author on reasonable request.
